# Therapeutic potential of TAM receptors in autoimmune diseases: insights from original studies

**DOI:** 10.1093/oxfimm/iqag001

**Published:** 2026-01-22

**Authors:** Sheu Ibrahim Adedayo, Taiye Abdullahi Gegele, Kehinde Ahmad Adeshina, Baliqis Adejoke Olukade, Ridwanullah Abiodun Abubakar, Adullateef Abdulsalam, Toheeb Oladejo Olalekan, Mohamed Mustaf Ahmed, Kasimu Ghandi Ibrahim

**Affiliations:** School of Medicine, Nazarbayev University, Astana, 010000, Kazakhstan; Department of Molecular Medicine, Morsani College of Medicine, University of South FL, Tampa, FL, 33612, United States; Department of Physiology, Wayne State University School of Medicine, Detroit, MI 48201, United States; Department of Physiology, Faculty of Basic Medical Sciences, Federal University of Health Sciences, P.M.B. 45, 751101, Nigeria; Department of Molecular Medicine, Morsani College of Medicine, University of South FL, Tampa, FL, 33612, United States; Department of Biomedical Sciences, Division of Physiology, Southern IL University School of Medicine, Carbondale, IL, 62702, United States; School of Medicine, Nazarbayev University, Astana, 010000, Kazakhstan; School of Medicine, Nazarbayev University, Astana, 010000, Kazakhstan; Faculty of Medicine and Health Sciences, SIMAD University, Mogadishu, 252, Somalia; Department of Basic Medical and Dental Sciences, Faculty of Dentistry, Zarqa University, P.O. BOX 2000, Zarqa, 13110, Jordan; School of Physiology, Faculty of Health Sciences, University of the Witwatersrand, 7 York Road, Parktown, Johannesburg, 2193, South Africa

**Keywords:** ligands (GAS6, Pros 1), therapeutic targets, biomarkers, remyelination, demyelination

## Abstract

TAM receptors, composed of Tyro3, Axl, and Mertk, belong to the receptor tyrosine kinase family and are activated by binding of their cognate ligands, Gas6 and Pros1. These receptor-ligand interactions mediate critical physiological processes, including the maintenance of immunological equilibrium, thrombocyte aggregation and subsequent thrombus development, apoptotic cellular debris clearance, homeostatic regulation of endothelial and vascular smooth muscle cells, and erythrocyte production. Perturbations in TAM signaling cascades have been shown to compromise the clearance of apoptotic cells, leading to persistent inflammatory responses that can contribute to the development of various autoimmune pathologies, including multiple sclerosis, rheumatoid arthritis, Sjögren’s syndrome, and systemic lupus erythematosus. We retrieved and reviewed only the primary studies addressing the roles of TAM receptors and their ligands in selected autoimmune diseases from Google Scholar, Scopus, Web of Science, and PubMed. The critical roles of TAM receptors in immune homeostasis and apoptotic cell clearance are well established. However, findings from several primary studies discussed in this review further emphasized that the loss of TAM receptor function in these processes significantly contributes to the pathogenesis and progression of autoimmune diseases. Herein, we highlight the role of TAM receptors in several autoimmune diseases, suggesting that TAM receptors are potential biomarkers for monitoring disease prognosis and therapeutic targets to improve patient outcomes.

## Introduction

TAM (Tyro3, Axl, and Mertk sometimes written as Mer) receptors are a subfamily of receptor tyrosine kinases activated by vitamin K-dependent Protein S (Pros1) and Growth Arrest-Specific Protein 6 (Gas6) ligands. [1] These receptors are found in cells of several systems, such as in the endothelial cells of blood vessels, phagocytic cells of the immune system, glia and neurons of the nervous and reproductive systems [2]. They play a crucial role in platelet aggregation and thrombus formation, apoptotic cell clearance, endothelial and vascular smooth muscle homeostasis, and erythropoiesis [3]. More importantly, they play a key role in immune response balance, particularly in inflammatory regulation [4]. As such, defect or downregulation of the TAM signaling pathway has been reported to contribute to inefficient apoptotic cell clearance, chronic inflammation, and autoimmune disorders, including multiple sclerosis, rheumatoid arthritis, Sjögren’s syndrome, and systemic lupus erythematosus [3, 5, 6].

Multiple sclerosis (MS) targets myelin sheaths in the central nervous system (CNS). This demyelinating cascade triggers a series of cellular events, including the compromised integrity of oligodendrocytes, extensive degradation of myelin architecture, and pathological activation of astrocytic populations, all culminating in neuronal damage [7, 8].

Systemic lupus erythematosus (SLE) is a disease characterized by a dysregulated immune system and the development of inflammation in multiple organs, mediated by immune responses [9]. A critical component of SLE pathogenesis involves impaired clearance of apoptotic cells, a process mediated by TAM receptor tyrosine kinases [10]. As a result, the accumulation of apoptotic debris in SLE patients has been postulated to contribute to the generation of autoantigens and the subsequent development of autoimmune responses [11].

Rheumatoid arthritis (RA) is a chronic disorder that affects the joints and leads to progressive damage and disability. The disease is characterized by a dysregulated immune response and chronic inflammation that result in morbidity [12, 13]. Despite its status as the predominant chronic inflammatory autoimmune arthropathy and reduced quality of life, RA has a diverse pathogenesis that is not yet completely understood. More so, Sjögren’s syndrome (SS), which is closely associated with RA and SLE, has been characterized by a dysfunction of the salivary and lacrimal glands, leading to systemic symptoms [14].

The critical role of TAM family members in immune regulation has been implicated in autoimmune diseases. These include SS, RA, MS, and SLE, where research has shown a decreased phagocytic activity and increased concentrations of soluble TAM receptors and their ligands in SS and SLE, suggesting that these factors may contribute to autoimmunity by impairing efferocytosis and presenting self-antigens [15, 16]. TAM receptors and their ligands, which are also expressed by oligodendrocytes, have been linked to oligodendrocyte stimulation and remyelination of neurons [17]. However, this review article provides a comprehensive analysis of seminal investigations examining the mechanistic implications of TAM receptor signaling and their corresponding ligands in autoimmune pathologies. This systematic evaluation elucidated the therapeutic potential of targeting the TAM axis as a novel approach for managing autoimmune disorders. This collective evidence underscores the significant promise of a TAM-directed therapeutic approach in expanding the available treatment for autoimmune diseases.

### Biology of TAM receptors

#### Mechanisms of TAM receptor activation and their role in cell signaling

TAM receptors have two identical immunoglobulin-like and fibronectin III repeats in the extracellular domain and a similar KIWAIES conserved sequence within their kinase domain [18, 19]. They are activated extracellularly by the endogenous ligands Pros1 and Gas6 [1]. Pros1 and Gas6 were gamma-carboxylated in the presence of vitamin K to achieve optimal activity. While Pros1 has a preference for Mertk and Tyro3, Gas6 interacts with all members, with the strongest affinity for Axl [20]. Furthermore, tubby and galactin-3 are other ligands recognized for TAM receptors. Although tubby and galactin-3 activate these receptors, Pros1 and Gas6 are natural and well-known ligands of TAM receptors. Galactin-3 and tubby binds to Mertk, whereas tubby-like (TULP1) protein interacts with Tyro3, Axl, and Mertk [21, 22].

Furthermore, Pros1 and Gas6 are linking molecules that connect T cells expressing TAM receptors with cells expressing phosphatidylserine (PS), such as aggregating platelets, apoptotic cells, and viral envelopes. The carboxy-terminus of the ligands interacts with the immunoglobulin repeats of the TAM receptors. In contrast, the amino-terminus interacts with the PS of expressing cells to establish a connection between the two cells [23]. Following TAM–ligand interactions, receptor homodimerization occurs, resulting in trans-autophosphorylation in the cytoplasmic kinase domain, leading to intracellular downstream signaling activation [24]. Also, interaction between Axl and other receptor tyrosine kinases (RTKs), such as epidermal growth factor receptor (EGFR), has been reported. This type of interaction is known as receptor heterodimerization and is independent of TAM ligands. This hetero-interaction has been suggested to regulate the expression of genes promoting cancer cell invasion [25, 26]. However, it has not yet been established whether hetero-interactions occur between Tyro3 and Mertk, as well as other RTKs.

The following intracellular pathways have been proposed to be stimulated following activation of TAM receptors: Nuclear Factor Kappa B (NFkB), Signal Transducer and Activator of Transcription (STAT), Focal Adhesion Kinase (FAK), p38 MAPK, the Mitogen-Activated Protein Kinases (MAPK/ERK 1/2 extracellular signal-regulated kinase), and the Phosphatidylinositol-3-Kinase (PI3K/Akt) and the Mammalian Target of Rapamycin (PI3K-Akt-mTOR) [22].

#### Molecular structures of TAM receptors, Gas6, and Pros1

**Figure 1:**
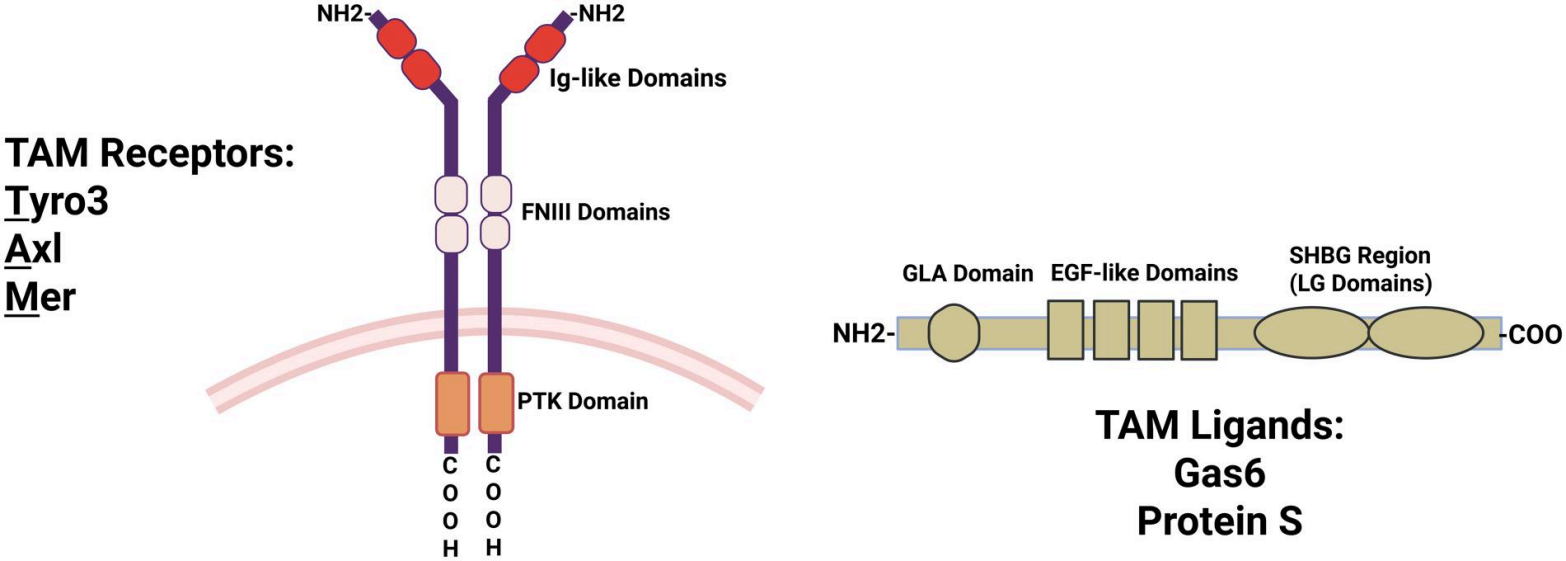
Molecular structures of TAM receptors Gas6 and Pros1. Phosphotyrosine kinase (PTK), Sex Hormone-Binding Globulin (SHBG), and gamma-carboxyglutamic acid (GLA) domains.

Tyro3, Axl, and Mertk have three distinct domains spanning the membrane [23]. They have an ectodomain with two immunoglobulin-like (IgL) and two fibronectin III repeats (FNIII); a single transmembrane domain; and a cytoplasmic domain (Fig. 1) [2, 27]. In humans, the kinase domain of each TAM member has three conserved tyrosine residues located as follows: Axl (Tyr698, Tyr702, and Tyr703), Tyro3 (Tyr681, Tyr685, and Tyr686), and Mertk (Tyr749, Tyr753, and Tyr753) [28, 29]. More so, the positions of tyrosine residues responsible for docking include: Axl (Tyr779, Tyr821, and Tyr866), Tyro3 (Tyr762, Tyr804, and Tyr828), and Mertk (Tyr847, Tyr872, and Tyr929) [22]. According to [18];

1. Transcripts encoded in the genes Tyro3, Axl, and Mertk ranged from 3 to 5kb.
2. Tyro3 and Axl encode 20 exons, while Mertk has 19 exons
3. Exons 2A, 2 B, and 2C of Tyro3 have alternatively spliced variants, indicating that these variants may have different functions, localizations, or undergo distinct posttranslational processes.
4. Splicing of exon 10 results in two alternative variants of Axl.
5. Tyro3 and Axl have molecular weights between 100 and 140kDa, and Mertk has a molecular weight between 165 and 205 kDa.
6. The amino acid residues in the full-length Axl, Tyro3, and Mertk were 894, 890, and 999, respectively.

Gas6 and Pros1 share a similar structure, featuring a C-terminal two globular laminin G-like (LG) domain, an epidermal growth factor (EGF-like) repeat domain, and an N-terminal gamma-glutamic acid domain (Gla, for carboxylation) [6]. TAM receptors and PS-expressing cells are connected via the carboxylated N-terminal glutamic acid residue, which interacts with the TAM receptors after the two globular LG domains bind the lipid PS on the membrane [30, 31].

The function and location of TAM receptors can be altered by proteolytic cleavage or the alternative splicing of gene transcripts. Metalloproteinases play a role in proteolytic cleavage of TAM receptor ectodomains, generating soluble decoy receptors. This affects the activation of these receptors, resulting in the loss of their functional roles [32]. ADAM9, ADAM10, ADAM17, and Furin are metalloproteinases recognized in the shedding of soluble TAM (sMertk and sAxl) receptors [33].

## Pathogenesis of autoimmune diseases

Autoimmune diseases are a collection of disorders characterized by abnormal B and T-cell responses to standard components of the body [34]. The immune system of the affected individual attacks its tissues [35]. These diseases are highly prevalent and affect persons of all age groups, particularly women [36, 37]. One of the most notable immunological manifestations is the production of autoantibodies, which serve as essential biomarkers for diagnosis, classification, and determination of the severity of the disease [34]. Autoimmune diseases typically arise from the interaction between environmental factors and genetic susceptibility. The most extensively characterized autoimmune pathologies include MS, SLE, RA, and SS.

### Pathophysiology of multiple sclerosis

MS is a devastating, chronic, neurodegenerative inflammatory disease that affects the CNS [38]. The underlying pathology is believed to be the damage to the myelin sheaths of the central nerves, likely caused by an autoimmune response [39]. This is corroborated by the discovery of plaques, which are regions of damage, specifically located within the white matter surrounding the lateral ventricles of the brain and optic nerves [40]. The demyelination of the white matter in MS is regularly observed using conventional MRI techniques [41]. However, lesions in the grey matter are also detected [42].

The pathogenesis of MS involves both cellular and humoral immunity [43, 44]. Active dendritic cells (DC) cause a shift in the balance of autoreactive T-cells (Th1, Th17), which, when differentiated, can then enter the CNS and initiate local inflammatory processes [45]. The inflammatory cascade initiates the secretion of proinflammatory cytokines, which subsequently compromises the integrity of the blood-brain barrier (BBB) and enhances its permeability. This disruption facilitates the transmigration of naïve T and B lymphocytes across the compromised BBB. Subsequent antigen presentation to T cells induces lymphocyte activation and differentiation, culminating in the expansion of both T helper cells (CD4+) and cytotoxic T cells (CD8+). These T cells can cause damage to myelin through both indirect and direct mechanisms. Indirect mechanisms include the induction of B-cell activation and antibody- or complement-mediated injury, whereas direct mechanisms involve cytotoxicity through degranulation (Fig. 1) [46, 47]. Moreover, neuropathological investigations have revealed distinctive patterns of white matter (WM) lesion formation, characterized by the significant involvement of T-lymphocyte infiltration and complement-mediated cascades in the process of myelin degradation (types I and II). Active multiple sclerosis (MS) lesions (types III and IV) have been associated with independent oligodendrocyte degeneration, which resembles viral or toxic components [48].

**Figure 2:**
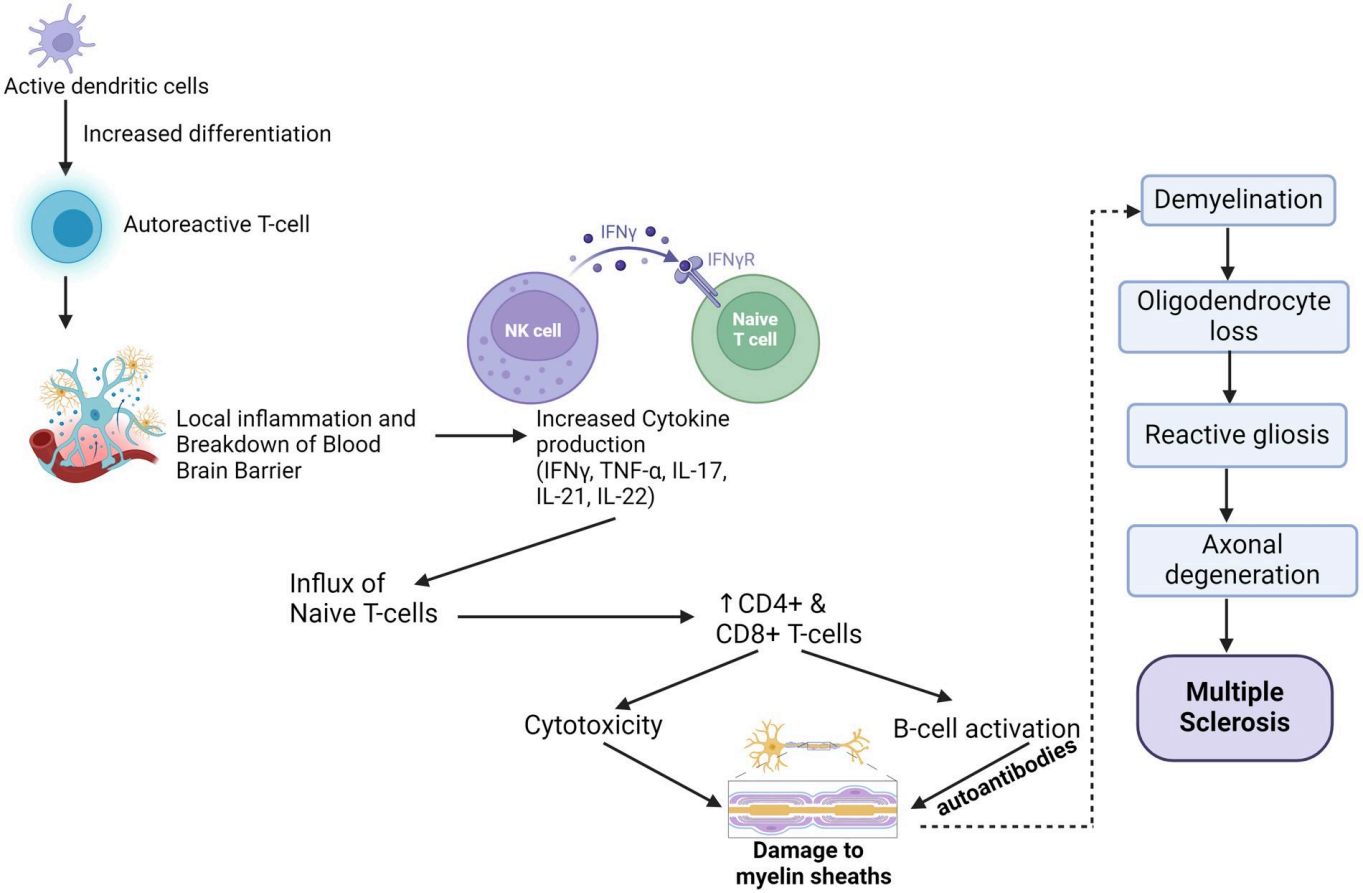
Pathogenesis of MS. Interleukin (IL); interferon gamma (IFNγ); tumor necrosis factor alpha (TNFα). Increased activation of CD4 + and CD8 + T cells, along with increased cytokine production caused by inflammation, leads to damage of myelin sheaths through cytotoxicity and B-cell activation.

The pathological process of MS involves collapse of the BBB, multifocal inflammation, loss of myelin, depletion of oligodendrocytes, reactive gliosis, and axonal degeneration (Fig. 2) [49]. MS has four neuropathological patterns based on the presence or absence of complement and immunoglobulins, apoptotic nuclei, and/or selective loss of myelin protein [48]. Relapsing MS patients frequently experience active lesions; however, these lesions are rare in progressive MS [50]. There are a high number of severed axons in acute multiple sclerosis (AMS) lesions, occurring roughly 12 times more often than in chronic lesions [51]. Therefore, axonal loss is seen during early disease onset and continues steadily. The transition from relapsing-remitting multiple sclerosis (RRMS) to secondary progressive multiple sclerosis (SPMS) is believed to occur when the brain reaches its limit in compensating for additional axonal loss [49]. Recent findings indicate that MS also affects the cortical and subcortical gray matter (GM), but to a lesser extent than white matter (WM) alterations [52, 53].

Although the pathophysiology underlying the progressive nature of this disease remains elusive, evidence suggests that hypoxia and energy-dependent axonal damage play crucial roles in this process. Proposed mechanisms contributing to this condition include mitochondrial dysfunction, oxidative stress mediated by reactive oxygen species (ROS) and nitric oxide (NO), disrupted redox homeostasis, neurotrophic factor deprivation, microtubule instability, ion channel dysfunction, and iron accumulation [54–58]. It has been suggested that meningeal inflammation and the formation of follicle-like structures generated by B-cells can increase the cytotoxic effects of SPMS [59]. Additionally, histological examinations conducted on individuals with progressive MS revealed a prominent presence of subpial cortical demyelination, as opposed to the significant occurrence of WM lesions in RRMS [60]. The question of whether GM harm is a direct consequence or a result of nearby WM disease has yet to be clarified.

### Pathophysiology of systemic lupus erythematosus

SLE is an autoimmune disease characterized by damage to multiple organ systems, including the hematologic, mucocutaneous, musculoskeletal, and renal systems. Pathogenesis is a complex interplay of factors and pathways, leading to varied clinical presentations [61]. The involvement of both innate and adaptive immunity can explain the basic pathology of SLE [62]. The pathogenesis of SLE involves a complex interplay of factors. These include impaired clearance of nucleic acids, an exaggerated type I interferon (IFN) response, compromised B-cell tolerance leading to autoantibody overproduction, immune complex formation and deposition, and subsequent multi-organ damage. Genetic susceptibility is a critical determinant, with multiple loci, including human leukocyte antigen (HLA) and non-HLA genes, contributing to disease risk. Ongoing research continues to identify novel susceptibility loci in diverse populations [63]. The disease initiation and perpetuation are influenced by genetic and environmental factors, as well as stochastic occurrences.

The pathogenesis of SLE is strongly linked to the excessive activation of various immune cells, including T cells, B cells, and monocytes, which exhibit aberrant methylation of differentially expressed genes [61, 64]. The presence of autoreactive T-cells in both human and murine models of SLE highlights an imbalance between pathogenic T cells and regulatory T cells (Tregs). Although CD4 + FoxP3+ Tregs have been extensively studied, recent research has emphasized the critical role of CD8 + Tregs (CD44 + CD122+Ly49+) in mediating lupus-like disease. This regulatory function is mediated by transcription factor Helios (TFH), a zinc finger protein belonging to the Ikaros family. Targeting Helios-expressing CD8 + T regulatory cells (Tregs) may represent a novel therapeutic strategy to promote self-tolerance and prevent SLE pathogenesis [65]. Paris-Muñoz *et al*. have discovered a decrease in the expression of Helios in CD8 + Tregs as lupus advances in mice [66].

**Figure 3:**
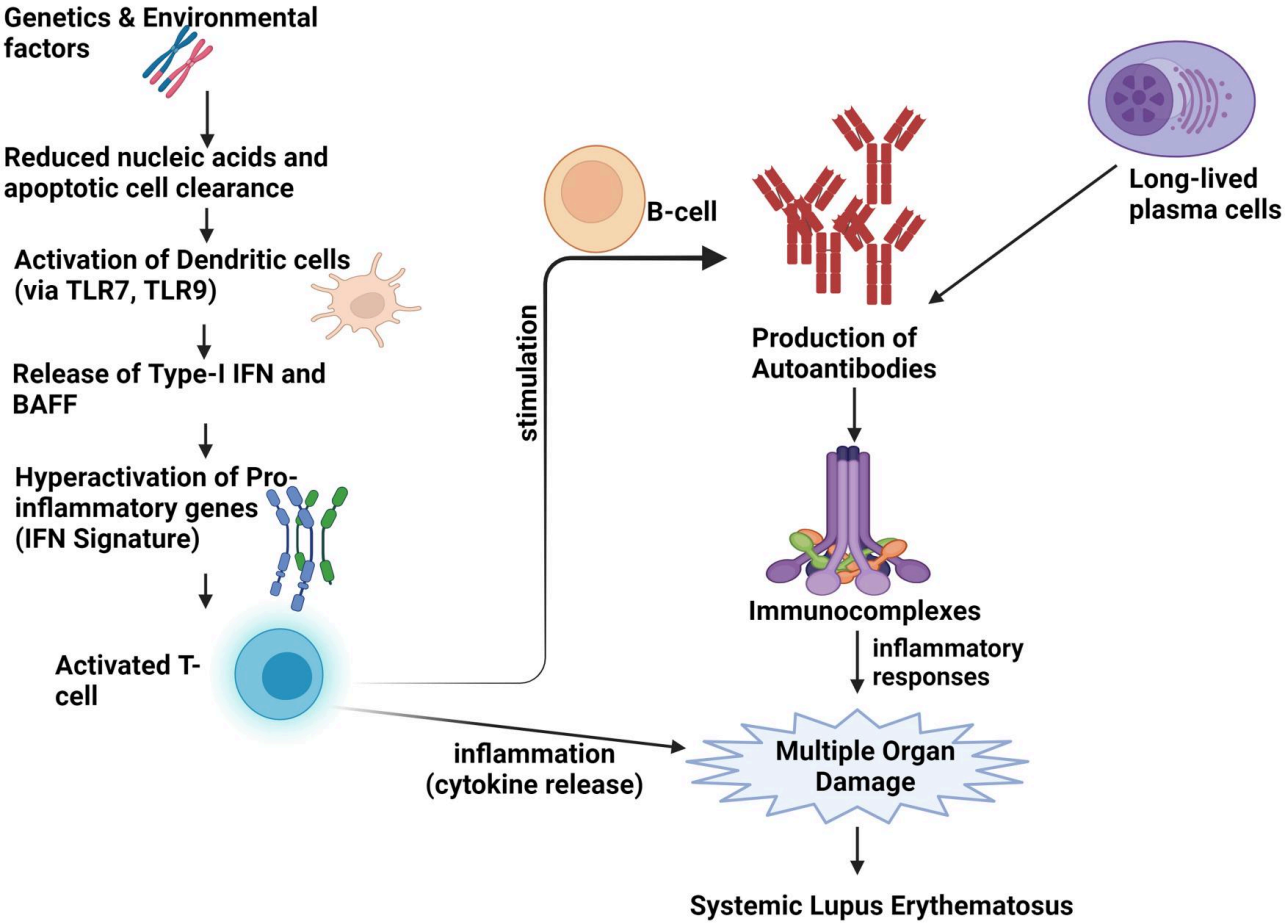
Pathogenesis of SLE. Toll-like receptors (TLR), interferon (IFN), and B-cell activating factor (BAFF). The progressive clearance of defective apoptotic cells leads to the activation of T cells, which in turn stimulates an increased inflammatory response, resulting in multiple organ damage.

In contrast to T cells, B cells play a pivotal role in the pathogenesis of SLE. In addition to antibody production against nuclear antigens, B-cells cells contribute to disease progression through antibody-independent mechanisms. These include antigen presentation, modulation of other immune cell functions, and cytokine secretion [61]. B-cells can be categorized into several subsets, including B1, B2, marginal zone (MZ) B-cells, and B-regulatory (B-reg) cells. B2 cells, also known as follicular B cells, generate targeted antibodies with high affinity against foreign antigens by collaborating with T cells in germinal centers (GCs). These cells also contribute to the development of immunological memory. MZ B-cells secrete IgM antibodies, while B1 cells produce germline-encoded “natural antibodies” that are mainly of low affinity and the multireactive IgM isotype [67]. These antibodies, which are typically components of innate immunity, are crucial for antimicrobial defence and cellular clearance, including apoptotic and necrotic debris. Dysregulated cell death pathways such as apoptosis and NETosis result in the release of autoantigens. These autoantigens are presented by follicular dendritic cells within GCs to autoreactive B cells, resulting in breakdown of self-tolerance. This ultimately results in the production of pathogenic autoantibodies, immune complex formation, and release of proinflammatory cytokines, which bind to certain nuclear self-antigens to form immunocomplexes that then precipitate in tissues. Ultimately, this process leads to inflammation and tissue damage in SLE (Fig. 3). A recent study has specifically examined the role of B1 cells in the development of SLE [68].

### Pathophysiology of rheumatoid arthritis

Rheumatoid arthritis (RA) is one of the most common, chronic, systemic autoimmune diseases characterized by the degradation of cartilage, formation of pannus, and proliferation of synovial tissue [69]. RA is further defined by the production of autoantibodies, such as rheumatoid factor (RF) and anti-citrullinated protein antibody (ACPA), as well as cartilage and bone deformities, synovial inflammation, and hyperplasia, which can lead to cardiovascular, pulmonary, psychological, skin, and skeletal disorders [70]. It could also lead to the development of systemic complications, which in severe cases can be life-threatening.

**Figure 4:**
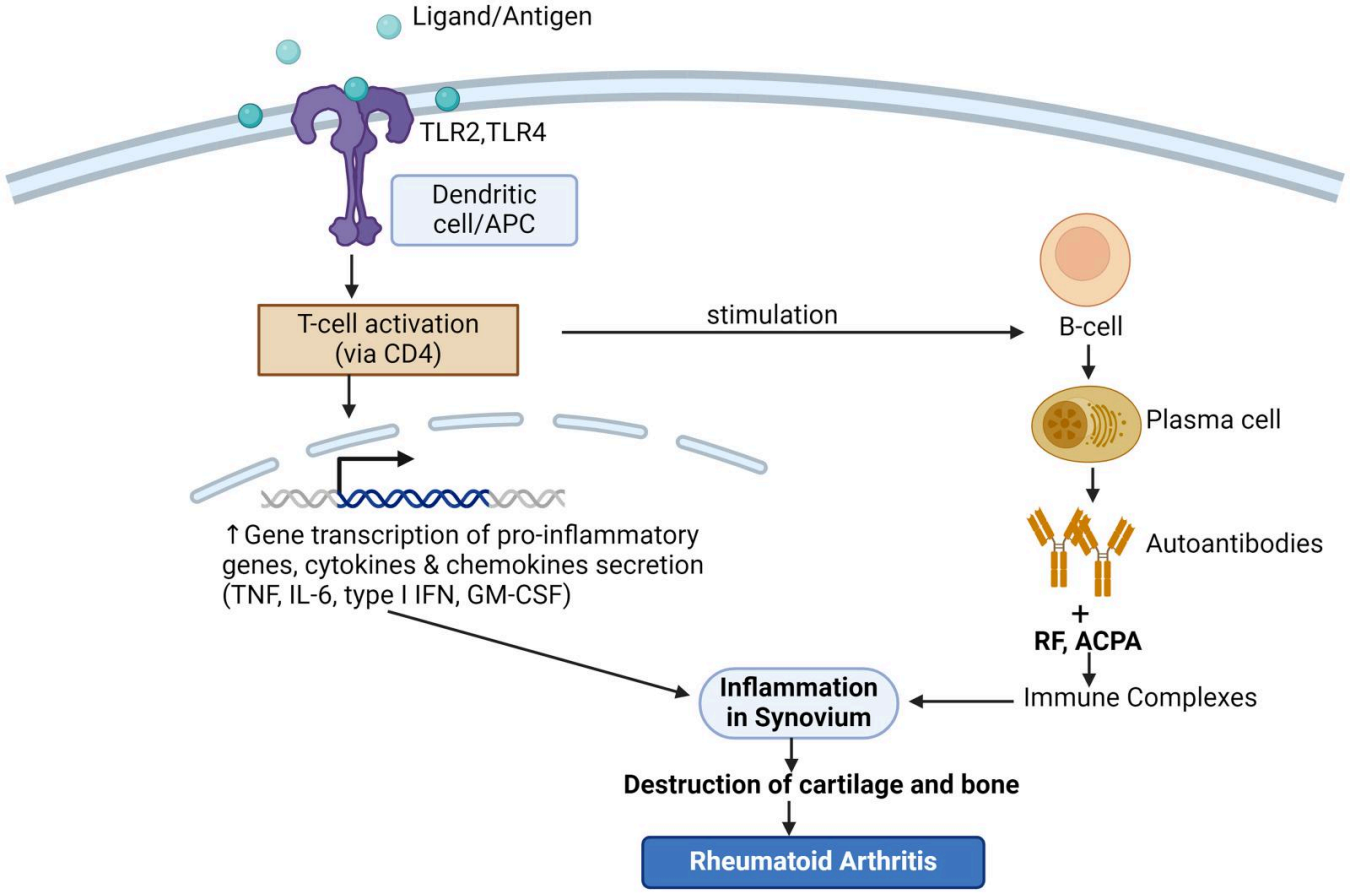
Pathogenesis of RA. Anti-citrullinated protein antibody (ACPA); Toll-like receptors (TLR); Interleukin (IL); Rheumatoid factor (RF); Interleukin (IL); Interferon (IFN); Granulocyte-macrophage colony-stimulating factor (GM-CSF). Activation of dendritic cells and T-cells leads to synovial inflammation and destruction of cartilage and bone, resulting from increased expression of proinflammatory genes and stimulation of B-cells.

Similar to other autoimmune diseases, T-cells, CD147, interleukins (ILs), and TNF-α are essential components of RA pathogenesis [71]. RA is considered pathologically heterogeneous [72]. The activation of innate immunity in the synovium by toll-like receptor (TLR) agonists or Fc receptor engagement is a critical pathogenic mechanism that results in inflammation during the early stages of RA [73]. Dendritic cells, monocytes, and macrophages are important components of innate immunity due to their roles in phagocytosis, antigen presentation, and cytokine synthesis. They also play a substantial role in the initiation and perpetuation of disease [74, 75]. Upon ligand binding, TLRs and Interleukin-1 receptors (IL-1Rs) initiate a cascade of intracellular signaling events that culminate in the activation of specific transcription factors. This process leads to the induction of gene transcription and the subsequent secretion of a variety of proinflammatory cytokines (e.g. TNF, IL-6, IFN, and growth factors such as GM-CSF) [76]. Nevertheless, caspase-1 must first process one of these cytokines, IL-1β, for it to be secreted. The inflammasome, a molecular complex activated upon detection of a danger signal, is necessary for the activity of caspase-1, a zymogen [77]. Gasdermin D (GSDMD) is also cleaved into two parts by caspase-1, specifically at the N- and C-termini. Cleavage of Gasdermin D (GSDMD) by inflammatory caspases releases its N-terminal fragment. This fragment oligomerizes within the plasma membrane, forming pores that allow the release of proinflammatory cytokines such as IL-1β and other cellular components. The released mediators act locally and systemically within the synovium, inducing an inflammatory response. This inflammatory response involves the activation and recruitment of various immune cells, ultimately contributing to the pathogenesis of RA (Fig. 4) [78].

Macrophages are key components responsible for the initiation and progression of RA [79, 80]. These cells are a significant source of degradative enzymes, chemokines, and cytokines, which stimulate joint inflammation and ultimately lead to bone and cartilage damage. Furthermore, it is also believed that macrophages are involved in synovial angiogenesis, which in turn plays a crucial role in the pathogenesis of RA [81]. The quantity of macrophages in the synovial tissue holds clinical significance as it serves as the most dependable marker for evaluating disease severity and the effectiveness of treatment. This is because the number of myeloid cells is directly linked to the inflammation of the synovial tissue in rheumatoid arthritis, as well as the progression of radiographic changes and disease activity [82]. To put it simply, the primary contribution of monocytes and macrophages to the development of RA is the secretion of free radicals, growth factors, proinflammatory cytokines and chemokines. Additionally, these cells release matrix metalloproteinases, which promote joint inflammation and damage [76].

In summary, RA progression is characterized by three distinct stages. These are a nonspecific inflammatory stage, which is further aggravated by T-cell activation in the synovium; a chronic inflammatory stage; and a tissue damage stage that is mediated by cytokines such as IL-1, IL-6, and TNF-α [83, 84].

### Pathophysiology of sjögren’s syndrome

SS is another chronic, complex, autoimmune, and female-predominant disorder often associated with ocular and oral dryness, resulting from the infiltration of mononuclear cells into the lacrimal and salivary glands [85]. It is also commonly referred to as 'autoimmune epithelitis’. However, the underlying cause of this condition remains unclear. Typically, both innate and adaptive immune responses are involved in the pathogenesis of SS, which can be triggered by viral infections and hormonal factors in genetically vulnerable individuals [86, 87]. It is believed that the interaction between genetic susceptibility and environmental triggering factors leads to the activation of the innate immune system during the initial phases of the disease. Over the past years, genetic studies have discovered more than 15 specific positions on chromosomes, known as loci, that are associated with SS [85]. It has been found that many of these loci are also linked to other autoimmune diseases, particularly SLE. SS is characterized by the excessive activation of B-cells by T-cells, progressing from asymptomatic states to the development of systemic complications and lymphoma [88]. At the tissue level, a characteristic feature is the infiltration of the salivary gland by lymphocytes, including B-cells, T-cells, and antigen-presenting cells [89] (Fig. 4). This is reflected in the essential diagnostic role of performing a biopsy of the minor salivary gland (MSG) [90]. B-cells have various potential roles in the development of diseases, including the production of autoantibodies, antigen presentation, and cytokine production [91]. B-cell hyperactivation is facilitated by genetic risk factors, as well as both T cell-dependent and independent mechanisms, and various subsets of pathogenic B-cells.

Salivary gland epithelial cells (SGEC) represent the focal point of disease-causing events in SS [92]. The pathological process of SS begins with the activation of epithelial cells, followed by alterations in the normal functioning of the glands. The disruption of glandular homeostasis occurs before lymphocyte infiltration. Emerging findings indicate that SGEC in SS are key players in the inflammatory and autoimmune response [93, 94]. The upregulation of MHC Class II, particularly HLA-DR, on salivary gland epithelial cells (SGEC) in regions with lymphocytic infiltration enhances their ability to present antigens to T cells as antigen-presenting cells. The presence of co-stimulatory factors CD80, CD86, and CD40 on salivary gland epithelial cells (SGECs) enhances their interaction with immune cells, leading to the release of Th1 cytokines [95]. Consequently, this creates a feedback loop that increases the expression of HLA antigens, co-stimulatory molecules, and adhesion molecules on SGEC [96]. SGECs produce chemokines, specifically CXCL13, CCL17, CCL19, CCL21, and CCL22, which facilitate the invasion of dendritic cells (DCs) [97]. IFNγ, a Th1 cytokine, also stimulates the synthesis of chemokines such as CXCL10 and CXCL9, which facilitate the migration of T-cells from the bloodstream to the salivary gland [98]. SGEC produce another chemokine called CXCL13, which guides the passage of B-cells into the salivary gland and facilitates the development of lymphoid tissues. SGEC secrete a diverse range of cytokines that play critical roles in both the innate and adaptive immune responses. This comprises interferons and other cytokines involved in Th1, Th17, and T follicular helper cell responses as well as B-cell activation.

**Figure 5:**
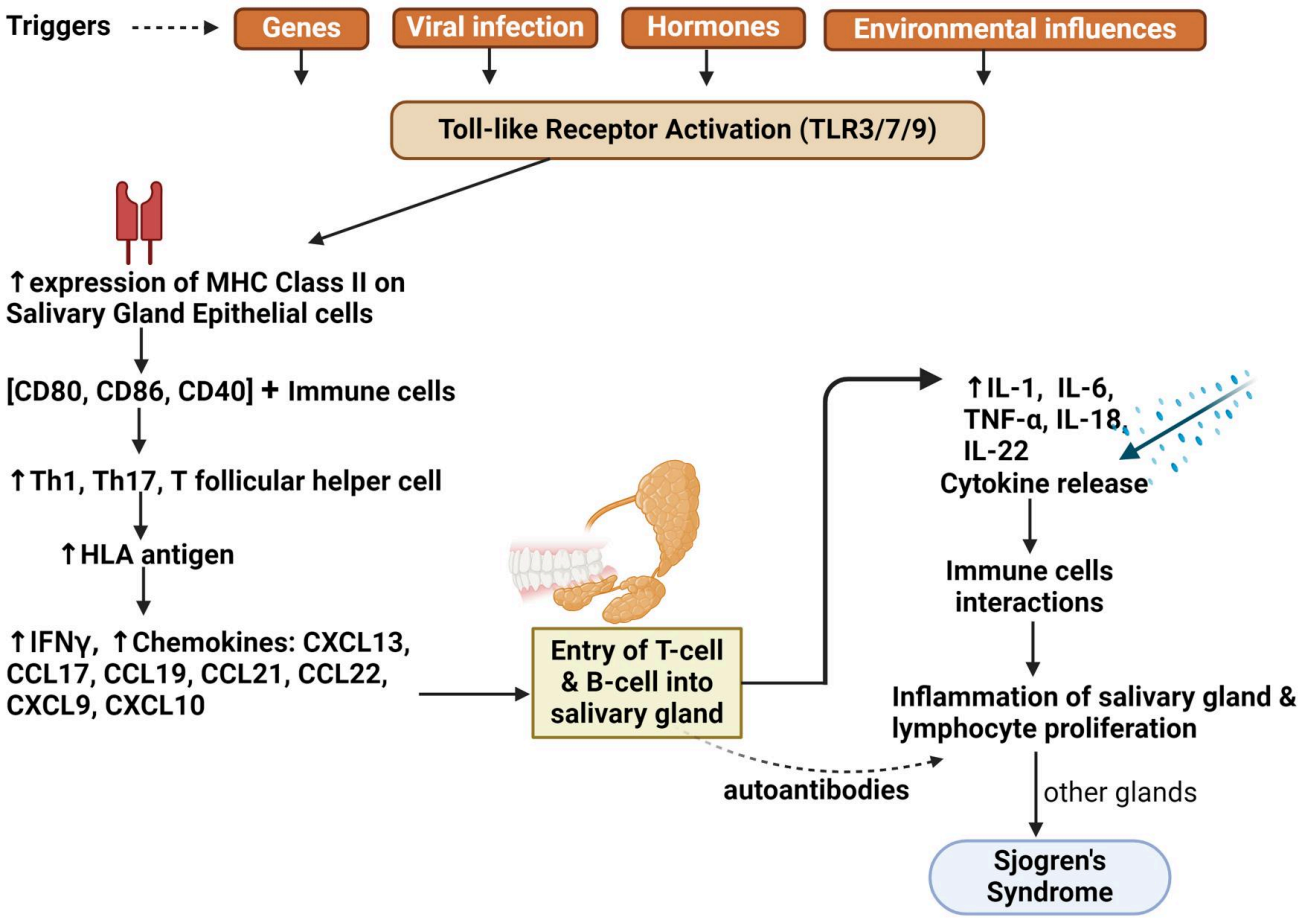
Pathogenesis of Sjögren’s syndrome. C-C motif ligand (CCL); Interleukin (IL); Interferon gamma (IFNγ); Tumor necrosis factor alpha (TNFα); C-X-C motif chemokine ligand (CXCL); Cluster of differentiation (CD); Th (T-helper); Human Leukocyte Antigen (HLA). Increased levels of cytokines, chemokines, and autoantibodies lead to salivary gland inflammation and lymphocyte proliferation.

Proinflammatory cytokines, such as IL-1, TNF, and IL-6, have consistently shown increased expression in salivary gland tissues of patients with primary Sjögren’s syndrome (pSS) [99]. The activation of IL-1 signaling in brain neurons has been previously hypothesized to be a crucial factor in the development of disease behavior, a condition that closely resembles chronic fatigue and is commonly observed in individuals with SS [100]. IL-33, a cytokine belonging to the IL-1 family, is elevated in both the serum and salivary gland biopsies of patients with SS [101]. It works together with IL-12 and IL-23 to stimulate the release of IFNγ by natural killer (NK) and NKT-cells [102]. There is a consensus that patients with pSS exhibit increased expression of genes controlled by both type I and type II interferons in both salivary tissue and peripheral blood. The overall consequence of excessive activation of immune and inflammatory responses in SS is impaired salivary gland function (Fig. 5).

## Roles of TAM and their ligands in autoimmune diseases

**Figure 6:**
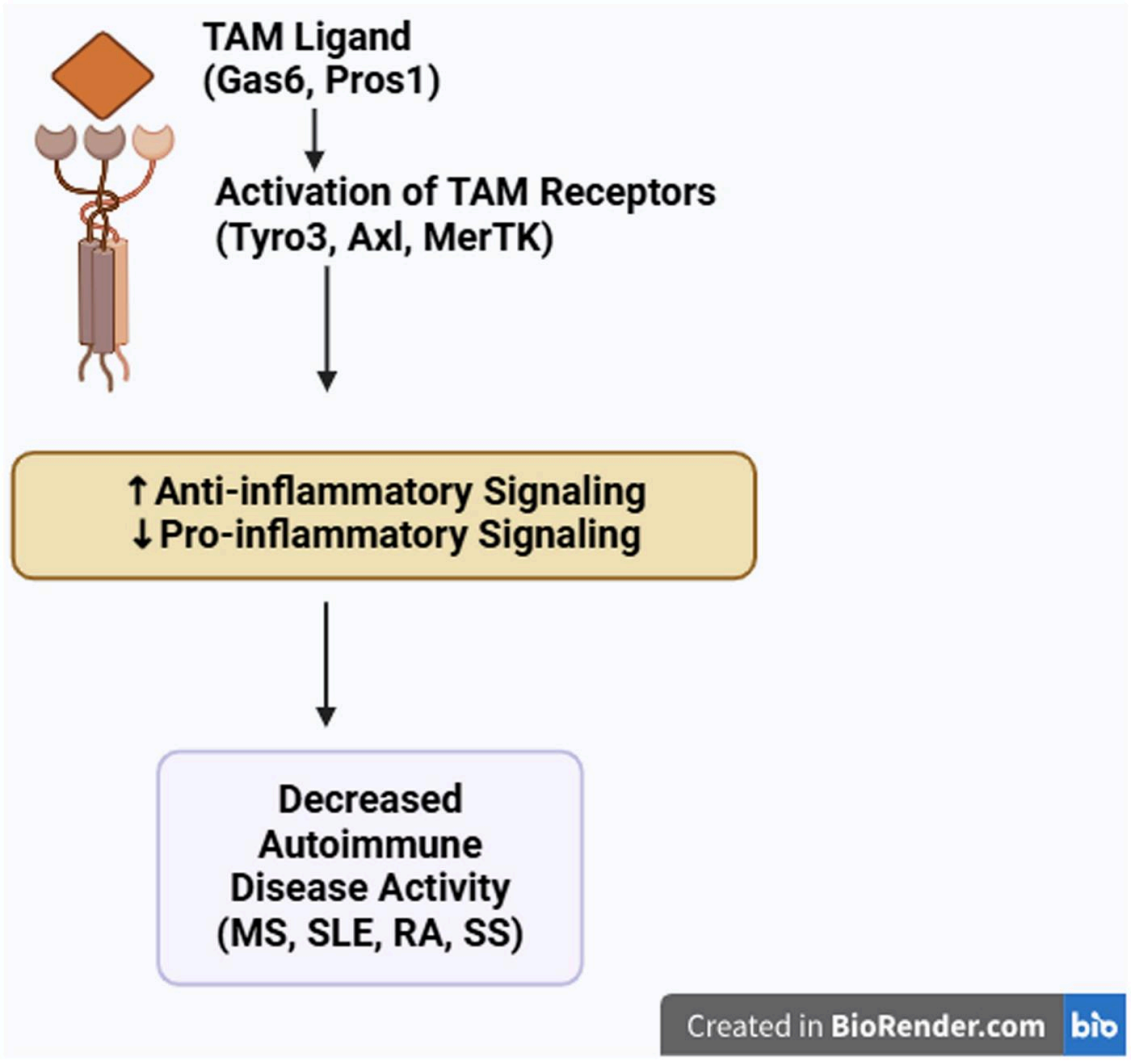
Summary of TAM receptor action in autoimmune diseases.

TAM receptor activation has been suggested to reduce pro-inflammatory responses and stimulate anti-inflammatory responses, thereby preventing autoimmunity [103]. This is one of the proposed mechanisms by which TAM receptors decrease disease activity (Fig. 6). We have discussed recent primary studies that have reported a role for TAM receptors and their ligands in MS, SLE, RA, and SS. There are limited studies on RA and SS; however, previous studies on MS and SLE have been highlighted in Tables 1 and 2.

**Table 1:** Summary of studies supporting a role for the Gas6, Pros1/TAM system in MS.

S/No	Type of Study	Findings	Authors
1	Consists of *in vivo* and *in vitro* components	Axl, Mer, and Gas6 expressions are higher following cuprizone-induced demyelination, while decreased Tyro3 mRNA is associated with oligodendrocytes. In Gas6 KO mice, demyelination is more severe than in control mice.	Binder *et al*. [111]
2	*In vivo*	Axl receptor tyrosine kinase deficiency resulted in a significant delay in the clearance of myelin debris and apoptotic oligodendrocytes following cuprizone-induced demyelination. This impairment in debris removal consequently delayed functional recovery and exacerbated axonal damage.	Hoehn *et al*. [112]
3	*In vitro*	There are elevated sAxl and sMertk levels in MS lesions, but these are not correlated with protective Gas6 levels, which may contribute to the prolongation of MS lesions.	Weinger *et al*. [113]
4	*In vivo*	Recombinant human Gas6 administration promotes remyelination following cuprizone-induced demyelination in mice.	Tsiperson *et al*. [114]
5	Consists of *in vivo* and *in vitro* components	Administration of exogenous Gas6 following cuprizone-induced demyelination in Gas6 KO mice enhances remyelination by oligodendrocytes in mice.	Binder *et al*. [115]
6		There is an association between SNPs within the Mertk gene and MS susceptibility.	Ma *et al*. [116]
7	*In vivo*	In Axl KO mice, demyelination and axonal damage are more significant than in WT during autoimmune EAE.	Weinger *et al*. [117]
8	Clinical study	Elevated CSF Gas6 levels are correlated with shorter MS relapses.	Sainaghi *et al*. [118]
9	*In vivo*	Intracerebroventricular Gas6 delivery in WT mice with EAE shows decreased axonal damage, less demyelination, and promotes neurofilament integrity; In Gas6 KO mice with EAE, increased axonal damage and inflammation are observed.	Gruber *et al*. [119]
10	*In vivo*	Mice with this loss of function in TAM activities exhibit a compromised capacity for clearing apoptotic cells across various tissues, increased concentrations of proinflammatory cytokines such as TNF-α and IL-6, and the generation of autoantibodies.	Tsou *et al*. [1]
11	*In vivo*	Mertk is identified as an MS susceptibility gene, which is differentially regulated in pathogenic CD4 + T-cells in EAE.	Hoppmann *et al*. [120]
12	*In vitro*	Total PROS1 in individuals with MS was decreased among MS patients in comparison to healthy individuals, which implied a possible modification in PROS regulation and proposed that reduced levels of circulating PROS might be linked to more adverse clinical consequences in MS, suggesting that PROS has the potential to function as a biomarker for assessing disease severity in MS patients.	Ma *et al*. [121]
13		Mertk upregulation, depending upon HLA-DRB1*15 : 01 status, is associated with increased or decreased risk of MS development.	Binder *et al*. [122]
14	*In vivo*	Gas6 and Axl knockout signaling leads to severe nerve damage, motor impairments, persistent inflammation, and hindered remyelination after cuprizone exposure. This suggests that Gas6-Axl signaling is essential for maintaining nerve health and regulating inflammation in the central nervous system, and its functions cannot be replaced by the Pros1/Tyro3/Mertk system.	Ray *et al*. [123]
15	*In vitro*	A decrease in the expression of Mertk and its ligand Gas6 causes a phagocytic deficit in macrophages derived from monocytes, resulting in a reduced ability to phagocytose myelin. At the same time, treatment with transforming growth factor-beta (TGF-β) can restore both the expression of Mertk and the phagocytic function of these macrophages, highlighting a potential therapeutic target.	Healy *et al*. [124]

**Table 2:** Summary of the previous studies supporting a role for the Gas6, Pros1/TAM system in SLE.

S/No	Type of study	Findings	Authors
1	Clinical study	Elevated sMertk and sTyro3 levels are observed in SLE patients. SLE patients show the highest level of sMertk compared to sTyro3 levels, and there was a significant association between sMertk levels and a higher SLE disease activity score and lupus nephritis	Wu *et al*. [138]
2	Clinical study	A positive association has been found between GAS6 polymorphism and cutaneous vasculitis in SLE patients. The prevalence of the GAS6 + 1332 T allele was higher in SLE patients with cutaneous vasculitis between the two Gas6 single-nucleotide polymorphisms (SNPs) (GAS6 834 + 7G/A and GAS6 + 1332C/T)	Wu *et al*. [139]
3	Clinical study	Decreased levels of Gas6 were observed in patients with class 1 lupus nephritis.	Gheita *et al*. [140]
4	Clinical study	Elevated serum Gas6 levels are found in SLE patients compared to controls, with a high specificity and moderate sensitivity for SLE diagnosis. However, Gas6 may serve as a reliable clinical marker for monitoring the progression of SLE disease and its response to treatment.	Kim *et al*. [141]
5	Clinical study	There is a correlation between sAxl and GAS6, whereas sMertk has been found to correlate with reduced Pros1. A strong correlation has been reported between disease activity indices, such as the Systemic Lupus Erythematosus Disease Activity Index and sMertk, compared with sAxl.	Zizzo *et al*. [16]
6	Clinical study	Elevated Gas6 serum levels have been found in SLE patients; decreased total and free Pros1 are found in the SLE patients compared to the control group, and the plasma concentration of the three soluble TAM receptors increases.	Recarte-Pelz *et al*. [142]
7	Clinical study	Elevated Gas6 serum levels have been found in SLE patients.	Wu *et al*. [143]
8	Clinical study	Elevated levels of sAxl are observed in the plasma of SLE patients, while mAxl is downregulated and Gas6 is slightly decreased in patients with SLE.	Zhu *et al*. [144]
9	Clinical study	Increased expression of Mertk in circulating monocytes and dendritic cells of patients with lupus, which may be enhanced by elevated IFN-I in SLE patients	Hilliard *et al*. [145]
10	Clinical study	mMertk and sMertk levels are significantly elevated in SLE compared to those in control subjects. While CD14 + CD16+ monocytes, which express anti-inflammatory Mertk cells, decreased in SLE.	Zhu *et al*. [145]
11	Clinical study	Gas6 levels are correlated with disease activity, and their levels increase significantly in the sera of patients with lupus nephritis and those with active SLE.	Bakr *et al*. [146]
12	Clinical study	Higher levels of sMer, sTyro, and sAxl were observed in JSLE patients, but a significantly decreased Mertk expression was observed on the monocytes of JSLE patients.	Ballantine *et al*. [15]
13	Clinical study	Higher levels of sTyro3, sAxl, sMer, and Gas6 ligands are observed in patients with active JSLE and nephritis.	Liphaus *et al*. [147]
14	Consists of *in vivo* and *in vitro* components	Matrix metalloproteases ADAM10 and TACE (ADAM17) are responsible for the cleavage of the ectodomain of membrane-bound Axl on the leukocytes of lupus-prone mice, as well as mediating cleavage of SLE PBMC Axl; loss of Axl on macrophages, both *in vitro* and *in vivo*, shows less anti-inflammatory signaling and exacerbates nephritis in the mouse disease model.	Orme *et al*. [148]
15	Clinical study	Higher serum levels of Axl were found in active SLE patients compared to inactive SLE patients; however, no correlation was observed between disease activity measures and levels of the TAM receptor or Gas6 ligand.	Mok *et al*. [149]

### Multiple sclerosis

The interplay between adaptive and innate immune responses fuels inflammatory damage to myelin in MS [104]. This involves myelin degradation and oligodendrocyte loss, primarily promoted by macrophages and microglial cells, as well as the release of proinflammatory cytokines. Previous research by Lu and Lemke showed that deleting just one TAM receptor caused minor effects, but losing two or all three receptors led to more severe problems [105]. These problems often included autoimmunity and chronic inflammation, particularly due to the accumulation of dead cells and the overactivation of immune cells. This suggests that difficulties in TAM signaling in specific immune cells may contribute to autoimmune diseases of the CNS.

In a mouse model of MS, activation of TAM receptors reduced the development of inflammation, which in turn decreased damage to nerve fibers. Additionally, in neurons that lack the Tyro3 receptor, the protective myelin layer surrounding the nerves becomes thinner and less organized. However, this does not necessarily affect nerve signal conduction in specific brain pathways [106].

In patients with MS who are also infected with parasitic worms (helminths), higher levels of TAM receptors on specific immune cells were linked to less severe inflammation, where it was observed that there was a decreased level of Th17 inflammatory reaction, which is due to the worsening of MS pathogenesis, suggesting that these receptors might play a protective role in MS [107]. According to Goudarzi *et al*. [108], microglia are the primary source of Gas6 in the CNS, and the anti-inflammatory role of Gas6 depends on its interaction with Tyro3 or Axl. In addition, Gas6 highly stimulates the anti-inflammatory/pro-repair cytokines interleukin 10 (IL-10) and transforming growth factor β (TGF-β), and IL-10 mediates the pro-myelinating action of Gas6 in the optic nerves.

Asadian *et al*. investigated how Gas6 plays a dependent role with the Tyro3 receptor in the initiation of remyelination. Tyro3 knockout (KO) in cuprizone models causes exogenous Gas6 to fail to promote the remyelination process and increases the number of myelinated axons in the corpus callosum. The study further reported that Gas6 acts directly on oligodendrocytes to promote remyelination rather than altering microglial activation [109]. The protective role of TAM receptors in the hindbrain of mice with autoimmune encephalomyelitis was reported, as the inhibition of TAM signaling caused a shift in the inflammatory response to the hindbrain parenchyma. This suggests that TAM signaling provides region-specific protection against inflammation induced by Th17 cells in encephalomyelitis (EAE) [110]. Key studies examining the Gas6, Pros1/TAM axis in multiple sclerosis are summarized in Table 1.

Moreover, higher levels of Gas6 in the blood are associated with less severe MS, suggesting that it could be a valuable marker for tracking disease progression. A Gas6-dependent role with the Tyro3 receptor provided this beneficial effect by specifically modifying the Gas6 molecule [125]. Additionally, when the Mertk receptor was knocked out in microglial cells (the brain’s immune cells), it led to a notable reduction in oligodendrocyte production during early development, resulting in the formation of pathological myelin where mice lacking microglial Mertk exhibited thinner myelin and showed impaired differentiation of oligodendrocyte precursor cells [126].

The Mertk-KO model highlighted an interrupted microglial reaction to demyelination, accompanied by a distinct demyelination-related oligodendrocyte pattern in both Mertk-wild-type (WT) and Mertk-KO mice, as revealed by single-cell RNA sequencing (scRNA-seq) [127]. Liu *et al*. [128] reported the beneficial role of Axl in hindering the progression of MS. In this study, dabrafenib, a potential therapeutic drug for the CNS, was beneficial for MS by increasing Axl expression. Techniques such as electroacupuncture have shown promise in animal models of MS by improving motor function and reducing demyelination, likely through the activation of Axl and Mertk receptors. Empirical evidence on the effects of electroacupuncture in a cuprizone-induced demyelination model on the role of TAM receptors (Axl and Mertk) in promoting recovery showed that electroacupuncture significantly improved motor coordinative dysfunction and reduced demyelination in treated mice, as evidenced by behavioral tests such as the beam-walking test and rotarod performance test [129]. The expression of Axl and Mertk was significantly upregulated following electroacupuncture, suggesting their involvement in therapeutics.

It has been demonstrated that TAM receptors play distinct roles in different tissues. According to a recent study, knockout of the Tyro3 or Gas6 gene reduced the severity of EAE in mice. The lymph nodes of the Tyro3 KO mice showed higher levels of both IL-17A and IL-4. In contrast, their spinal cord tissues express lower levels of both IL-17A and IL-4 [130]. The use of betulin and clofibric acid, which are Axl-targeting compounds, emphasized the significant role of the receptor in alleviating EAE. *In vitro*, both drugs enhanced phagocytosis, promoted M2 polarization, inhibited apoptosis, improved mitochondrial structure, delayed the S phase of the cell cycle, and reduced inflammation. While EAE was reported to be alleviated *in vivo*, Axl gene deletion reversed this effect [131]. According to a recent in silico study, antisense oligonucleotides designed to target Axl gene transcripts show promising potential in reducing MS symptoms [132]. These results further implicate the Axl receptor in the pathogenesis of MS. A single-nucleotide polymorphism analysis of the MerTK gene indicates that patients with the MerTK rs7422195 SNP GA/AA are associated with a greater risk of radiological disease activity, according to a retrospective study conducted on MS patients who had previously received natalizumab medication [133].

### Systemic lupus erythematosus

TAM receptors are expressed in macrophages and are involved in macrophage efferocytosis, a process that involves clearance of apoptotic cells. Zhou *et al*. [134] reported elevated serum levels of IgG-type autoantibodies against Tyro3 in SLE patients compared to those with RA, pSS, and HCs. This suggests that IgG-type autoantibodies may be involved in SLE pathogenesis by targeting Tyro3 on macrophages and consequently impairing efferocytosis. Several studies have investigated the levels in SLE, examining their correlation with disease activity and specific clinical parameters. Liphaus *et al*. found that sMertk levels were significantly higher in patients with active JSLE than in those with inactive JSLE and healthy controls. The increased levels of sAxl and sMertk were influenced by glucocorticoid treatment. In contrast, decreased sTyro3 levels were observed following glucocorticoid treatment. This study further reported that sAxl levels are also correlated with disease activity; however, sMertk has a stronger correlation, indicating that greater disease activity and inflammation are associated with higher sMertk levels. However, the exact roles of sTyro3 and Gas6 are not established in this study, emphasizing the need for further investigation [10].

Biomarkers for a specific disease are crucial because they help monitor disease progression and effectiveness of interventions for the disease. This is because there is always a connection between the biomarkers and disease activity. TAM receptors are recognized for their potential as biomarkers for various diseases, and their roles in pathogenesis and progression have been well documented. For instance, Soliman *et al*. [135], suggested a connection between plasma Axl concentration and SLE activity. The study further reported a higher plasma concentration of Axl in patients with active SLE and active renal SLE than in those with inactive SLE and active non-renal SLE. The elevated levels of Mertk receptor on NK cells in SLE patients, when compared with those in HCs, emphasize the clinical relevance of this receptor in disease activity, with the Mertk gene being one of the dysregulated genes in NK cells [136]. In both RAW264.7 cells and pristine-induced lupus mice, hydroxychloroquine enhances efferocytosis via the Mertk/Gas6 signaling pathway, underscoring the importance of this pathway as a therapeutic target [137]. A summary of clinical and experimental studies assessing the Gas6, Pros1/TAM signaling as potential biomarkers and therapeutic targets in systemic lupus erythematosus is presented in Table 2.

### Rheumatoid arthritis

Van den Brand [150] demonstrated that administering Pros1 and Gas6, either locally or systemically, to activate TAM receptors, can potentially decrease proinflammatory signaling and modulate the adaptive immune response, thereby reducing arthritis severity (Fig. 5). Additionally, overexpression of Pros1 has been shown to extensively activate Mertk in the synovial joints, leading to the resolution of arthritis [151]. Several studies have proposed TAM receptors as potential targets for alleviating the symptoms of RA. However, improper activation can lead to adverse effects. For instance, Waterborg *et al*. [152] demonstrated that administration of Mertk receptor agonistic antibodies can be detrimental by blocking efferocytosis in arthritic joints. This study also highlighted the protective role of Mertk in joint inflammation, where Pros1 significantly contributes to Mertk activation and the subsequent reduction in disease severity. The alteration in the plasma concentration of Gas6 and Axl in RA patients suggests the involvement of the receptor and its ligand in the RA pathogenesis because decreased levels of Axl and Gas6 are significant in RA patients [153].

Furthermore, a joint-specific protective role has been attributed to Axl and Mertk, primarily because of their differential expression levels in the synovium of various joints. For example, in an RA mouse model, Axl KO and Mertk KO mice showed exacerbated arthritis pathology in the ankle and knee joints, respectively. In contrast, Tyro3 KO mice did not exhibit any protective or aggravating effects in either the ankle or knee joints [154].

In another study, Axl was prominently expressed in ankle synovial cells, and this expression was enhanced by TGF-β1 [155]. The decreased Axl expression significantly contributed to the worsening of arthritis pathology observed in the ankle joints of mice following Axl gene deletion. More recently, Nerviani *et al*. [156] demonstrated that the histopathology of synovial tissue in naïve patients with RA is correlated with Axl levels, as elevated Axl levels are associated with decreased resident immune cells, reduced disease activity, and lower expression levels of inflammation-related genes. The study further suggested a connection between sAxl levels in the synovial fluid and synovial histopathology. These findings highlight the impact of Axl expression on the outcome of inflammatory arthritis. The expression of Mertk and Axl can be affected by the stage of RA and treatment, specifically IL-6 expression. Individually, TAM receptors play distinct roles in the pathogenesis of rheumatoid arthritis (RA), as shown in an antibody-induced arthritis model using K/BxN serum administration [157]. In this study, Axl and Mertk KO mice exhibited severe development of arthritis with a marked increase in inflammatory cytokines, whereas Tyro3 KO mice showed minimal joint inflammation and a decreased inflammatory response.

Additionally, triple TAM KO mice, lacking all three receptors, developed early stage arthritis characterized by bone marrow edema [152]. The role of TAM receptors in the immune response and pathogenesis of RA has been further highlighted by studies comparing their expression levels between RA and osteoarthritis (OA), a non-autoimmune disease. Zheng *et al*. [158] that the expression levels of TAM receptors, particularly Axl and Mertk, were significantly higher in the synovial tissue of RA patients compared to those of OA patients. The study further reported elevated levels of soluble TAM receptors (sTAMs) in the synovial fluid of RA patients. These findings suggested a strong association between increased TAM receptor levels and RA pathogenesis. In addition, Wang *et al*. [159] examined the effects of Tyro3 on fibroblast-like synoviocytes (FLS) and found that blocking Tyro3 *in vitro* led to decreased production of inflammatory cytokines, reduced matrix metalloproteinase activity, and fewer autoimmune-promoting helper T-cells. Additionally, the study showed that Tyro3 KO resulted in reduced synovial inflammation, reduced joint damage, and improved immune cell balance.

Furthermore, elevated Gas6 levels have been reported following arthritis induction. The study also observed higher bone mass in a single Tyro3 KO (Tyr3^-/-^) mouse model of arthritis than in their wild-type littermates. In addition, less synovial hyperplasia, osteoclast numbers and bone damage were observed in Tyro3 KO (Tyr3^-/-^) compared with controls [160].

The clinical relevance of TAMs has primarily focused on their potential as therapeutic targets or biomarkers in diseases where their roles have been established. In patients with RA, Tyro3 expression is highly significant in CD14 + monocytes and these expression levels are clinically relevant for measuring disease activity. Axl shows a low level of expression, and Mertk has no significant expression level in RA [161]. The study suggests that Tyro3 is a potential therapeutic target and biomarker for RA.

### Sjögren’s syndrome

The improper removal of apoptotic cells has been proposed as a mechanism for the emergence of autoimmunity in SS. When dead cells are phagocytosed through TAM receptors, intrinsic suppression of inflammation occurs; however, dysfunction in this process can lead to the development of autoimmunity [162]. High peripheral blood levels of Type I IFN and the activation of IFN-stimulated genes are additional characteristics of SS, supporting the role of TAM signaling failure in SS development [163, 164]. A study by Qin *et al*. [165] reported decreased mRNA levels of Axl and Tyro3 in primary Sjögren’s syndrome (pSS) patients, whereas the sMer levels increased in their plasma. The study further reported a positive correlation between sMer levels and Sjögren’s Syndrome Disease Activity Index.

Moreover, Gas6 has been suggested as a risk factor for pSS, as evidenced by lower plasma Gas6 concentrations in patients than in controls [166]. They further reported a significant association between an increased risk of SS and decreased plasma Gas6 levels. Witas *et al*. [167] emphasized the role of TAM receptors, particularly Mertk, in SS by investigating the role of Mertk signaling in a murine model of SS. Following Mertk knockout, the study found that mice developed decreased salivary flow, anti-nuclear autoantibodies (ANA), apoptotic cells, and lymphocytic infiltrates in the submandibular gland (SMG). In addition, decreased Mertk signaling and increased sMertk, which corresponds to higher ADAM17 activity, were observed in SS-susceptible mice [167]. However, these data suggest that Mertk plays a protective role in SS, consistent with the role suggested for Mertk in other autoimmune disorders. More recently, the clinical relevance of Mertk and Tyro3 in the minor salivary glands of patients with SS was evaluated. Lee *et al*. [168] reported an association between Mertk and laboratory features of SS, organ damage, and clinical disease activity. Therefore, Mertk level could be a relevant marker for predicting patient prognosis.

Recently, the evaluation of expression levels of TAM receptors in minor salivary gland tissues of SS patients shows that only the Mertk receptor was significantly associated with higher focus scores, anti-SS-A antibody titers, serum IgG levels, and increased EULAR Sjögren’s Syndrome Disease Activity Index and Damage Index scores [169].

## Discussion, future direction and conclusion

Evidence from both clinical and preclinical studies suggests a role for TAM receptors in autoimmune diseases, making TAM signaling a critical target for intervention. Tyro3 and its ligand, Gas6, among other members, have significant contributions to MS pathogenesis. Additionally, the proremyelination effects of Tyro3 and Gas6 can be attributed to their signaling in oligodendrocytes. For example, Tyro3 knockout (KO) in cuprizone models causes exogenous Gas6 to fail to promote remyelination, resulting in an increase in the number of myelinated axons in the corpus callosum. The study further reported that Gas6 acts directly on oligodendrocytes to promote remyelination rather than altering microglial activation [109]. However, a tissue-specific role has been suggested for TAM receptors. As reported by Binder et al. [130], knockout of the Tyro3 or Gas6 genes reduced EAE severity in mice, with increased expression levels of both IL-17A and IL-4 in the lymph nodes of Tyro3 KO mice and decreased expression levels of both IL-17A and IL-4 in spinal cord tissues. Thus, a more thorough investigation is needed to clarify the tissue-specific roles of TAM receptors, which could help to identify patients with multiple sclerosis who are more likely to benefit from TAM-targeted treatments. Soluble TAM receptors hold promise as biomarkers for monitoring disease activity, and clinical studies have demonstrated their correlation with disease activity and clinical parameters. A connection between plasma Axl concentration and SLE activity has further implicated Axl in the pathogenesis of SLE. More so, it should be noted that studies on SLE are primarily clinical; however, research should also focus on *in vitro* and animal studies to gain mechanistic insights into the individual role of TAM receptors in SLE at the molecular level. Moreover, TAM receptors present both opportunities and challenges as therapeutic targets for RA. Receptor-specific and stage-specific effects caution against a one-size-fits-all approach despite preclinical data indicating their protective role in reducing inflammation and joint destruction. The distinct roles of Tyro3, Axl, and Mertk in various synovial microenvironments and disease stages need to be clarified in future research. These findings will be crucial for developing safe and focused treatments and for demonstrating that TAM molecules are accurate biomarkers for RA. Finally, as studies investigating the role of TAM receptors in SS remain limited, future research should emphasize this area.

In conclusion, the collective evidence highlights the important role of TAM receptors, Tyro3, Axl, and Mertk, and their ligands in initiating and perpetuating autoimmune conditions, such as MS, SLE, RA, and SS. Although clinical and preclinical research highlights their therapeutic and diagnostic potential, the evidence also reveals complex tissue-specific and receptor-specific roles that need to be interpreted with caution. Tyro3 and Gas6 have pronounced pro-remyelination activities in MS, and soluble TAM receptors, particularly Axl, are good candidates for use as biomarkers to measure disease activity in SLE. However, inconsistencies between model systems and human trials suggest that further mechanistic research, primarily *in vitro* and animal models, should be undertaken to reveal their precise molecular functions. In addition, the differential effects of TAM receptors in different tissues and disease stages of RA and other autoimmune diseases require context-specific focused therapeutic interventions. However, enlarging the scope of research into TAM receptor biology is essential for the creation of precise, safe, and effective diagnostic and therapeutic approaches to autoimmune diseases.
